# 

**Published:** 1911-06

**Authors:** 


### CONTENTS.

ORIGINAL COMMUNICATIONS

A Preliminary Report; Etiology, Pathology and Treatment of Pellagra; a new Theory, a Specific cause. George C. Mizell, M. D. P. H. D., Atlanta, Ga. ........ 111

Treatment of Pulmonary Tuberculosis by Artificial Pneumothorax, Showing Author's Apparatus. Dr. S. T. Harris, Valdosta, Ga. ........ 125

Psychotherapy in Habitual Constipation. E. T. Gibbs, M. D., Gainesville, Ga. ........ 138

Tubal Pregnancy. J. R. B. Branch, M. D. ........ 150

Some Observations about the Alimentary Canal. J. C. Brock, Carrollton, Ga. ........ 153

The Value of the Pathologist to the Surgeon. Dr. E. C. Davis, M. D., Atlanta, Ga. ........ 155

Hemorrhage in Typhoid Fever. Dr. C. W. Strickler, Atlanta, Ga. ........ 158

Bacilli Carriers and their Relation to Public Health. K. R. Collins, M. D., Atlanta, Ga. ........ 161

"An External Urethrotomy". Frank P. Norman, M. D., Greenville, Ga. ........ 166

EDITORIALS ........ 168

NEWS AND NOTES ........ 169

